# Supplementary material for: Manifestations of muscle fatigue in baseball pitchers: a systematic review
Source: PeerJ. 2019 Jul 29;7:e7390. doi: 10.7717/peerj.7390 (PMC6673423; doi:10.7717/peerj.7390)
Supplement: Supplemental Information 1 [file peerj-07-7390-s001.docx]

**Statement of Rationale and Contribution of the work.**

**Manifestations of muscle fatigue in baseball pitchers: a systematic review**

**1. The rationale for conducting the meta-analysis**

It has been well documented that fatigue in baseball pitchers is linked to lowered physical and mental performance, injury, and changes in kinematics. Numerous studies have associated fatigue with overuse, high ball velocities, lack of rest time, poor mechanics, and degree of self-satisfaction. However, there is a lack of conclusive evidence in the literature on the relationship between performance outcomes, pitcher kinematics and kinetics as a result of fatigue. To our knowledge, there has not been a systematic review of the literature on the effects of fatigue on baseball pitchers. This work will systematically review the literature to identify a theoretical framework for the relationship between outcomes and the manifestation of fatigue on baseball pitching.

**2. The contribution that the meta-analysis makes to knowledge in light of previously published related reports, including other meta-analyses and systematic reviews.**

Our results demonstrate a co-dependence between changes in kinematics and a decrease in performance. This stems from central and peripheral fatigue, which is a primary reason for injury in baseball pitchers. A large percentage of baseball pitchers exhibit pain or soreness in either their elbow or shoulder, or both at some point in a season. Initially, kinematic changes occur that could maintain performance, but increase joint loading. Performance decreased with elevated pitch counts and innings thrown, and pitching further into games or the season led. Evidence was found to be consistent across all studies. With a proof of concept established, the prevention of negative outcomes associated with fatigue must be the focus of future research and performance should not be the only criteria.
